# The prevalence of peripheral arterial disease in diabetic subjects in south-west Nigeria

**DOI:** 10.4102/phcfm.v4i1.354

**Published:** 2012-10-03

**Authors:** Bolaji O. Oyelade, Akintayo D. OlaOlorun, Louis O. Odeigah, Isaac O. Amole, Olufemi S. Adediran

**Affiliations:** 1 Lautech Health Centre, Ogbomoso, Nigeria; 2 Bowen University Teaching Hospital, Nigeria; 3 University of Ilorin Teaching Hospital, Nigeria; 4 Benue State University Teaching Hospital, Nigeria

## Abstract

**Background:**

Peripheral arterial disease (PAD) is rarely sought for and generally under-diagnosed even in diabetics in developing countries like Nigeria. PAD is easily detected and diagnosed by the ankle-brachial index, a simple and reliable test.

**Objectives:**

To determine the prevalence of PAD in diabetic subjects aged 50–89 years and the value of ankle-brachial index measurement in the detection of PAD.

**Method:**

A cross-sectional descriptive study of 219 diabetic subjects aged 50–89 years was carried out. The participants were administered a pre-tested questionnaire and measurement of ankle-brachial index (ABI) was done. The ankle-brachial index < 0.90 was considered equivalent to peripheral arterial disease.

**Results:**

The overall prevalence of PAD was 52.5%. The prevalence of symptomatic PAD was 28.7% whilst that of asymptomatic PAD was 71.3%. There were a number of associations with PAD which included, age (*p* < 0.05), sex (*p* < 0.05), and marital status (*p* < 0.05). The use of the ankle-brachial index in the detection of PAD was clearly more reliable than the clinical methods like history of intermittent claudication and absence or presence of pedal pulses.

**Conclusion:**

The prevalence of PAD is relatively high in diabetic subjects in the south-western region of Nigeria. Notable is the fact that a higher proportion was asymptomatic. Also the use of ABI is of great value in the detection of PAD as evidenced by a clearly more objective assessment of PAD compared to both intermittent claudication and absent pedal pulses.

## Abstrait

**La prévalence des maladies artérielles périphériques chez les sujets diabétiques dans le sud-ouest du Nigéria**

**Contexte:**

Les maladies artérielles périphériques (MAP) sont rarement recherchées et généralement sous-diagnostiquées même chez les diabétiques dans les pays en développement comme le Nigéria. Les MAP sont facilement détectables et diagnostiquées par l'indice de pression tibio-brachiale, un test simple et fiable.

**Objectifs:**

Déterminer la prévalence des MAP chez les sujets diabétiques âgés de 50 à 89 ans et la valeur de la mesure de l'indice de tibio-brachiale dans la détection de maladies artérielles périphériques.

**Méthode:**

Une enquête transversale descriptive sur 219 sujets diabétiques âgés de 50 à 89 ans a été réalisée. Un questionnaire pré-testé a été remis aux participants et la mesure de l'indice tibio-brachial (ITB) effectuée. Un indice tibio-brachial inférieur à 0.9 était considéré comme indiquant une maladie artérielle périphérique.

**Résultats:**

Le taux de prévalence des MAP était de 52.5%. La prévalence des MAP symptomatique était de 71.3%. De nombreuses associations avec les MAP ont été constatées, notamment l’âge (*p* < 0.05), le sexe (*p* < 0.05), le statut marital (*p* < 0.05). L'utilisation de l'indice tibio-brachial dans la détection de maladies artérielles périphériques était manifestement plus fiable que les méthodes cliniques telles que des antécédents de claudication intermittente ou la présence de pouls pédieux.

**Conclusion:**

La prévalence des MAP est relativement plus importante chez les sujets diabétiques dans la région du sud-ouest du Nigéria. Il faut souligner le fait qu'une grande proportion était asymptomatique. En outre, l'utilisation de l'ITB est d'une grande valeur dans la détection des MAP comme en témoigne une évaluation plus objective des MAP comparée à la fois à la claudication intermittente et à l'absence de pouls pédieux.

## Introduction

### Key focus

Peripheral Arterial Disease (PAD) is an important cause of ischemic limb, delayed wound healing and lower extremity amputation in diabetic patients. There is significant lack of awareness of this condition by many physicians, and it therefore remains under-diagnosed and underestimated. The prevalence of PAD worldwide has been estimated at between 4.5% and 29%.^1^ The prevalence of PAD in diabetics in sub-Saharan Africa varies from 1.7% to 28%.^2^ In our Nigerian environment there is a paucity of studies to assess its prevalence especially in diabetics. Agaba^3^ at the Jos University Teaching Hospital found that the prevalence of PAD in type 2 diabetics type 2 with end stage renal failure was 51.7%.

In 2005, an estimated 1.1 million people died from diabetes mellitus. The incidence of diabetes mellitus is showing an alarming rise in developing countries. According to the World Health Organization (WHO), almost 80% of deaths from diabetics occur in low and middle income countries, and the WHO projects that deaths due to diabetes will increase by more than 50% by 2015 without urgent action.^4^

Epidemiologic evidence has shown that there is a strong association between diabetes and PAD. Peripheral Arterial Disease is defined as a partial or complete obstruction of one or more arteries usually of the pelvis or lower limbs, caused by atherosclerosis.^5^ It may be asymptomatic or may manifest as symptoms of compromised blood flow with exercise (mainly intermittent claudication) or in severe cases, at rest. Peripheral arterial disease affects a large portion of the adult population worldwide.^4^

As many as 27 million people are estimated to have PAD in North America and Europe. Approximately 800 000 Canadians are affected with PAD, about 4% of those over the age of 40 and 20% of those over the age of 75.^6^ More than 50% of diabetics have obvious atherosclerotic cardiovascular disease within 15 years of the onset of diabetes and are 20 times more likely to have significant lower extremity arterial disease than non-diabetics.^7^

The ankle-brachial index (ABI) is the ratio of the ankle and the brachial systolic blood pressure and is used to assess individuals with PAD. An ankle-brachial index < 0.90 suggests the presence of PAD and is a marker of cardiovascular risk.^8^ The ankle-brachial index is an objective, simple, reliable and non-invasive way of detecting PAD and it is determined by blood pressure measurements being taken at the arms and ankles using a Doppler. The ankle-brachial index test is simple enough to be performed as an office or consulting room procedure and apart from being one of the most reliable tests for PAD, it is also not invasive and the least expensive.^7^

The relatively high prevalence of Doppler-diagnosed vascular lesions (18% – 28%) contrasts with the low clinical (absence of pulses) prevalence of peripheral vascular disease (4.4% – 8.2%).^3^ This implies that several individuals will remain undiagnosed if only clinical assessment was relied upon to diagnose PAD and it explains the value of the ankle-brachial index in the assessment of PAD. The ankle-brachial index is 95% sensitive and 99% specific for PAD compared to an angiogram.^9^ The angiogram is the gold standard for the diagnosis of PAD, however it is invasive and associated with other complications like allergic reaction to the contrast medium, thrombosis and embolism and it is not always available. The ankle-brachial index on the other hand is not invasive, has no complications, is readily available and could be learned within a short time. So the use of the ankle-brachial index in the assessment of PAD is of very high value.

### Objectives

The study was to determine the prevalence of PAD in diabetic subjects aged 50–89 years and the value of the ankle-brachial index measurement in the detection of PAD. Clinical assessment (intermittent claudication and presence or absence of pedal pulses) on the other hand could not detect the majority of the asymptomatic subjects with PAD.

### Significance of the study

The primary care physician is strategically positioned to help in the early detection of PAD and thus able to institute management to prevent further worsening of the condition, otherwise gangrene with subsequent amputation and other cardiovascular complications becomes almost inevitable. Since most patients are asymptomatic and carry potentially significant morbidity and mortality risks, screening for PAD should become a routine practice at primary care level.

## Ethical considerations

The Ethics Committee of the Baptist Medical Centre, Ogbomoso (now Bowen University Teaching Hospital) granted approval for the study. Informed consent was also obtained from the participants before commencement of the study.

### Methods

The study was a cross-sectional prospective survey that was started in October 2009 and was completed in April 2010. The study population consisted of adult male and female diabetic subjects aged 50–89 years who were seen at the medical outpatients department. Two hundred and nineteen subjects were recruited for this study. Others were those who had an amputation from non-diabetic causes, those with foot deformities, and those with known hemoglobinopathies. Through a systematic sampling method, every second patient was recruited for the study. Subjects below 50 years of age, subjects who are older than 89 years, non-diabetic subjects, subjects with past or current history of smoking, and subjects who withheld consent for the study were excluded. The number of patients attending the medical outpatients’ clinic was estimated to be approximately 100 per day. About 15 fell into the age range of 50–89 years. The diabetic clinic was run for 3 days every week giving a total of about 45 patients every week. So, a sampling fraction of 2 was taken and a simple random sampling was done to pick the first patient from the first two patients as the starting number of the systematic sampling technique and subsequent selections were every second patient registered.

An identification sticker was placed on all selected patients’ record cards by the researcher with the assistance of the records officers and then the selected patients’ cards were sent to a designated consulting office for the study. The selected patients were screened and those who met the inclusion criteria were recruited for the study after they gave a written informed consent. The identification sticker was left on all patients’ cards until the completion of the study to avoid a repeat selection. A pre-tested questionnaire was administered to obtain the demographic data. Other information obtained from the questionnaire included: history of intermittent claudication, previous foot ulceration or amputation, leg pain at rest and examination to detect presence or absence of the pedal pulses. The pre-testing of the questionnaire was done with thirty subjects during which necessary adjustments were made to the questionnaire before the commencement of the study. Subjects with symptomatic PAD were those who had intermittent claudication and had PAD (i.e. ankle-brachial index < 0.90) whilst those with asymptomatic PAD did not have intermittent claudication but had PAD (i.e. ankle-brachial index < 0.90).

### Setting

Ogbomoso is located about 100 km north of Ibadan, capital of the Oyo State in southwest Nigeria. The indigenous people belong to the Yoruba ethnic group, who engage mostly in farming or trading. There are two renowned academic institutions in Ogbomoso – Ladoke Akintola University of Technology and the Nigerian Baptist Theological Seminary – that attract people from other ethnic groups to the city. A government-owned general hospital, a Baptist hospital, a few primary health care centres and an increasing number of private hospitals meet the health needs of the people. The hospital, where the study was carried out, is a 200-bed faith-based hospital that renders primary and secondary health care. It is the referral centre for all other hospitals in Ogbomoso.

## Procedure

### Protocol for ankle-brachial index

The same protocol was used for all patients. The ankle-brachial index was obtained using the mercury sphygmomanometer made by Dekamet Accosson, England with appropriate cuff size for each subject and an 8.1 MHz hand-held Doppler device made by Parks Medical Electronics, Aloha, Oregon, USA. The cuff (about 12.5 cm wide) of the sphygmomanometer was applied evenly and snugly about the bare arm with the lower edge at 2.5 cm above the ante-cubital fossa. A cuff of about 15 cm wide was used for all obese subjects. The subject was made to rest quietly in the supine position for a minimum of five minutes before readings were taken in the supine position.

The brachial artery was palpated and identified. Light application of the Doppler gel on the skin was done for ease of sound transmission and the Doppler probe was held at an angle of about 50 degrees to the artery to ensure that the best quality of signal was obtained.^10^ The arm pressure was determined by applying the blood pressure cuff with the lower edge at 2.5 cm above the ante-cubital fossa and inflated to at least 30 mmHg above the level at which radial pulsation disappeared so as to ensure complete collapse of the brachial artery. The Doppler probe was held at the brachial artery just distal to the cuff. The cuff deflation proceeded slowly at no greater than 2 mmHg per second. As the cuff was being deflated slowly the investigator listened for the first korotkoff sound with the Doppler probe placed over the brachial artery in the ante-cubital fossa. The pressure was measured in both arms, three times in each arm and the average was found, the higher average pressure of the two was taken and recorded.

At each ankle, the posterior tibial artery was palpated, the cuff was applied at about 2 cm above the ankle and inflated to at least 30 mmHg above the arm systolic pressures, thus ensuring complete collapse of the posterior tibial artery. Cuff deflation proceeded slowly, no greater than 2 mmHg per second. The pressure was taken at the point at which the first Doppler signal appeared during deflation of the cuff. This was done three times and the average was determined. The observed value was recorded to the nearest 2 mmHg. The systolic pressure only was used for the determination of the ankle-brachial index.

The average ankle pressure was divided by the average higher Doppler-determined brachial artery systolic pressure and this gave an ankle-brachial index (ABI) for each leg, hence there was right ABI and left ABI. The normal range for ABI is 0.9–1.3.^11^

The data analysis was done with the Epi-Info statistical software (version 3.4.1).

## Results

A total of 219 subjects were recruited for the study, and consisted of 90 males and 129 females. The male to female ratio was =1:1.4 and the ages of the subjects ranged from 50 to 89 with a mean age of 63 ± 8.76 years.

The prevalence of PAD assessed through the use of ABI was 52.5% (115 subjects), whilst when the assessment was done with the use of a history of intermittent claudication, the prevalence was 24.7% (54 subjects). Also the prevalence of PAD was found to be 11.4% (25 subjects) (*p* > 0.05) when absent pedal pulses were employed for diagnosis. (Table 1)

**TABLE 1:** Peripheral Arterial Disease (ABI < 0.9) Intermittent Claudication (IC) and pedal pulses.

Variables	*f*	%
**PAD**		
Present	115	52.5
Absent	114	47.5
Total	219	100
**Intermittent Claudication (IC)**		
Present	54	24.7
Absent	165	75.3
Total	219	100
**Pedal pulses**		
Present	194	88.6
Absent	25	11.4
Total	219	100

*f*, Frequency.

**FIGURE 1:**
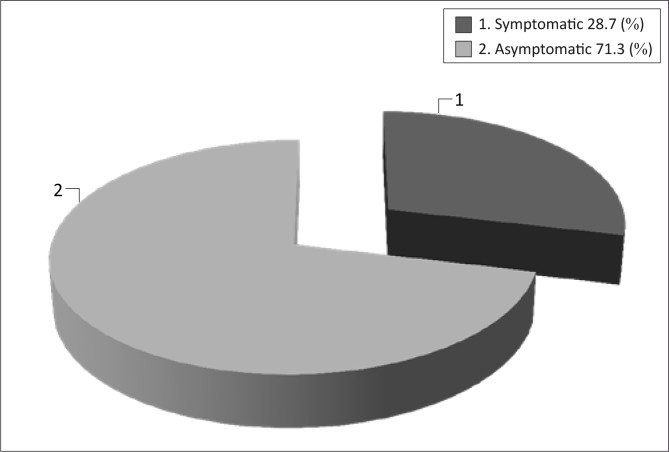
Symptomatic and asymptomatic peripheral arterial disease.

The prevalence of symptomatic PAD was 28.7% whilst that of asymptomatic PAD was 71.3% (Figure 1). The females were more in number 129 (58.9%) subjects than males (Table 2).

**TABLE 2:** Distribution of subjects by socio-demographic characteristics.

Variables	*f*	%
**Age Range (years)**		
50–59	83	37.9
60–69	76	34.7
70–79	49	22.4
80–89	11	5.0
**Sex**		
Male	90	41.1
Female	129	58.9
**Marital Status**		
Divorced	2	0.9
Married	156	71.2
Widowed	61	27.9
**Educational Level**		
No Formal Education	111	50.7
Primary	38	17.3
Secondary	17	7.8
Tertiary	26	11.9
University	27	12.3

*f*, Frequency.

**TABLE 3:** Association between age, sex, marital status and pad.

Variables	PAD		Normal		Total		*p*-value	*χ*^2^
					
	*n*	%	*n*	%	*n*	%		
**Age Group (years)**								
50–59	45	44.1	57	55.9	102	100	0.010	11.44
60–69	41	56.2	32	43.8	73	100	-	-
70–79	23	62.2	4	37.8	37	100	-	-
80–89	6	85.7	1	14.3	7	100	-	-
**Sex**								
Male	42	46.7	48	53.3	90	100	0.076	2.09
Female	73	56.6	56	43.4	129	100	-	-
**Total**	**115**	**52.5**	**104**	**47.5**	**219**	**100**	**-**	**-**
**Marital status**								
Married	71	45.5	85	54.5	156	100	0.004	10.98
Divorced	1	50	1	50	2	100	-	-
Widowed	43	70.5	18	29.5	61	100	-	-
**Total**	**115**	**52.5**	**104**	**47.5**	**219**	**100**	**-**	**-**

*χ*^2^, Chi-square; *n*, Given as number; PAD, Peripheral Arterial Disease.

The majority of the subjects were married and their spouses were alive (72.1%). One hundred and eleven (50.7%) subjects had no formal education and those with University education accounted for only 12.3%. The prevalence of PAD amongst males was 46.7% whilst the prevalence of PAD amongst women was 56.6%.

The occurrence or development of PAD seemed to increase with age (*p* < 0.05). Forty-five (44.1%) of the subjects within the age range of 50–59 years had PAD whilst 6 (85.7%) subjects of those in the age range of 80–89 years had PAD. Forty-three (70.5%) subjects out of 61 of those who were widows or widowers had PAD (Table 2). The number of subjects with intermittent claudication increased as age increased too except for a slight decrease in the age range 70–79 years and *p* < 0.05 (Table 4).

**TABLE 4:** Association between intermittent claudication, age group, and sex.

Variables	Intermittent Claudication (IC)				Total		*P*-value	*χ*^2^
					
	Present		Absent					
					
	*n*	%	*n*	%	*n*	%		
**Age Group (years)**								
50–59	12	14.5	71	85.5	83	100	0.03	8.89
60–69	23	30.3	53	69.7	76	100	-	-
70–79	14	28.6	35	71.4	49	100	-	-
80–89	5	45.5	6	54.5	11	100	-	-
**Sex**								
Male	19	21.1	71	78.9	90	100	-	-
Female	35	27.1	94	72.9	129	100	-	-
**Total**	**54**	**24.7**	**165**	**75.3**	**219**	**100**	**-**	**-**

*χ*^2^, Chi-square; *n*, Given as number; IC, Intermittent Claudication.

## Discussion

In this study, it was statistically significant that intermittent claudication (IC) increased with age (Table 4) except for a slight decrease in the 70–79 age range. It was found that 16.7% of those in the age range 50–59 years had IC, whilst 57.1% of those in the age range 80–89 years had IC. A similar finding was reported in the data from subjects in the Rotterdam population study.^5^ In the Rotterdam study it was found that the prevalence of symptomatic PAD increased from 1% in the age range 55–59 years to 5% in the age range 85–89 years. The reason for the higher prevalence in this study might be because the current study was hospital-based whilst the Rotterdam study was a population-based study. The second reason is that diabetic patients alone were recruited for this study unlike the Rotterdam where less than 20% were diabetic subjects.^12^

The overall prevalence of PAD in diabetic patients aged 50–89 years was 52.5% in this study. A similar finding was reported by Agaba^3^ at the Jos University Teaching Hospital, where he looked at the characteristics of type 2 diabetics, presented with end stage renal failure and found the prevalence of PAD to be 51.7%. However other studies have reported a lower prevalence in sub-Saharan Africa and some other parts of the world. Kengne, Amoah and Mbanya^2^ in their report on the cardiovascular complications of diabetes mellitus in sub-Saharan Africa (Cameroon, Ghana, Kenya and Tanzania) declared that the prevalence of PAD in diabetic patients varies from 1.7% to 28%. This implies that the prevalence of PAD varies widely from place to place.

Kengne, Amoah and Mbanya noted the difference in prevalence between the use of ankle-brachial index (which gave higher prevalence 18% – 28%) and the use of clinical (absent pulses) examination (which gave lower prevalence 4.4% – 8.2%). In the current study, it was also found that if absent pulses were used to make the diagnosis of PAD, the prevalence of PAD would have been 11.4% (Table 1) compared with the prevalence of 52.5% with the use of the ankle-brachial index. This demonstrates the value of the ankle-brachial index in the detection of PAD. The use of only absent pedal pulses would have been very unreliable. There is paucity of data in Nigeria and sub-Saharan Africa on the prevalence of PAD in diabetics.

The prevalence of PAD found in this study was quite high compared to some other studies reported above. This may be due to the fact that the majority of our diabetic patients in this environment present late, often with complications^13^ when the progression of peripheral arterial disease had probably gone unchecked. It should be noted that tight control of blood glucose leads to delay in and sometime prevention of some long term complications like atherosclerosis and nerve damage and should be attended to by primary care physicians.

It was reported that of those patients with PAD, over one-half are asymptomatic or have atypical symptoms, about one-third have claudication, and the remainder have more severe forms of the disease.^14^ In this study about 71% of those found to have PAD were asymptomatic and less than one-third were symptomatic (had intermittent claudication). The use of the ankle-brachial index in the assessment of peripheral arterial disease in this study clearly demonstrated the higher prevalence of asymptomatic PAD. Previously when intermittent claudication (IC) was relied upon to diagnose PAD, several cases were missed and from this study only 28.7% of those who actually had PAD would have been diagnosed if IC had been used as the diagnostic method. It should be realised that in contrast to PAD in non-diabetic individuals, PAD is more prevalent in diabetic subjects, and because of the distal territory of vessel involvement and its association with peripheral neuropathy, it is more commonly asymptomatic.^15^

In the current study, the prevalence of intermittent claudication in those patients who had PAD, was 28.7%. As noted above, diabetic patients are more prone to be asymptomatic, hence with the use of intermittent claudication (or use of symptoms) less than 50% of the diabetic subjects will be diagnosed with PAD and in the current study it was 28.7% as earlier reported. Therefore a more objective and reliable tool of assessment for PAD is indispensable.

Those who were widows or widowers had a higher prevalence of PAD than those who were married. Seventy and half per cent of those who were widows or widowers also had PAD (Table 2). A possible reason for this finding may be because those who are married enjoyed the support of their spouses, such support could be financial, psychological and spiritual. It is important to note that the primary care physician can easily learn how to use the hand-held Doppler to diagnose PAD and institute management early to prevent possible loss of limb and reduction in quality of life that might result if undetected.

## Limitations of the study

There was no treadmill to do a functional testing in subjects with claudication. Subjects with claudication will typically exhibit a 20 mmHg drop in ankle pressure after exercise. It is recommended to make use of a treadmill in future research.

## Recommendations

The Ankle-brachial index is recommended annually for diabetic subjects to screen for the presence of peripheral arterial disease. More studies need to be carried out in Nigeria on PAD in diabetic subjects, for example, it will be very appropriate to study PAD in subjects aged 50–89 years who do not have diabetes mellitus.

## Conclusion

The findings from this study suggest that the prevalence of PAD in diabetic patients presenting at the health care facility in Ogbomoso is high, and higher amongst women and is associated with age and sex.

The use of the Doppler determined ankle-brachial index has shown the reliability and objectivity in detecting peripheral arterial disease especially in the asymptomatic subjects (who constituted 71.3% of those diagnosed to have PAD), who would not have been detected through other methods like the use of history of intermittent claudication and absent pedal pulses. It is the hope of the researchers that family physicians and others who care for the diabetic patients will embrace the regular use of Doppler determined ABI in their various services to the diabetic population. It is easy to learn within a short time and it is not expensive.
